# Supplementary figures and images for: From sabers to spikes: A newfangled reconstruction of the ancient, giant, sexually dimorphic Pacific salmon, †Oncorhynchus rastrosus (SALMONINAE: SALMONINI)
Source: PLoS One. 2024 Apr 24;19(4):e0300252. doi: 10.1371/journal.pone.0300252 (PMC11042722; doi:10.1371/journal.pone.0300252)

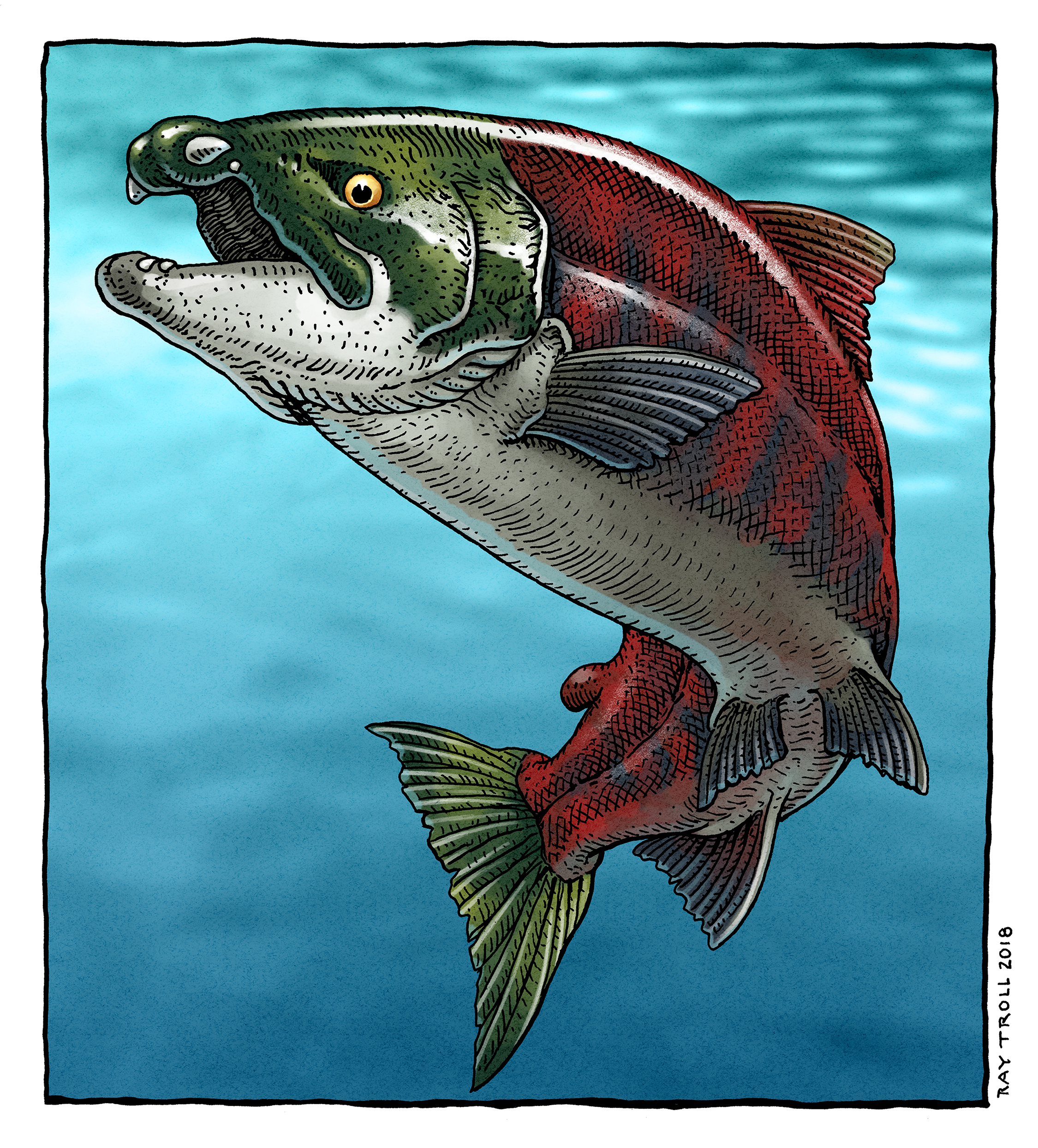

Supplement: S1 Fig — (TIF) [file pone.0300252.s003.tif]
