# Supplementary material for: From sabers to spikes: A newfangled reconstruction of the ancient, giant, sexually dimorphic Pacific salmon, †Oncorhynchus rastrosus (SALMONINAE: SALMONINI)
Source: PLoS One. 2024 Apr 24;19(4):e0300252. doi: 10.1371/journal.pone.0300252 (PMC11042722; doi:10.1371/journal.pone.0300252)
Supplement: Attachment — Submitted filename: ResponseToReviewers_PONE-D-23-29788.docx [file pone.0300252.s004.docx]

Response to reviewers and editor comments:

To Reviewer1: Thank you. We are excited to share these outcomes with the broader scientific and public communities.

Reviewer 2:
This is a fine and detailed reassessment of an amazing fish species. The techniques are appropriate. The descriptive anatomy is good and the illustrations are necessary and appropriate. I have only minor suggestions.

1. I am not sure what the authors mean by the term "salmonins." Their systematic section classifies the genus within the subfamily Salmoninae, but the correct/standard way to informally refer to that clade is "salmonines"
If they mean to refer to a tribe or subtribe, that would require yet another spelling.

**Response: These instances were corrected to salmonine(s).**

2. The extended discussion of possible functions seems to me to be a bit over the top in terms of detail, and the language used is rather informal compared to the rest of the manuscript. I think it could be shortened by about a quarter and revised to a more technical style. I also noticed that some but not all of the in-text citations use the author-date style whereas most of the manuscript uses numbered citations.

**Response: A more technical style was implemented and as a result, we reduced the text in that section by nearly a quarter. The author-date style citations were corrected to follow the Vancouver format.**

3. Throughout my reading I noticed occasional problems of punctuation (missing or incorrect) and rarely missing words. Mostly it's well written. I left it to the authors and copy editor to find and fix those in the final draft.

**Response: We each read the manuscript again to find as many of these instances as possible.**

4. I am sceptical of the idea that these huge fishes were semelparous, even though their relatives were and are. I can't think of many examples of huge animals that breed only once. Is there any way to determine (growth rings on vertebrae, otoliths, ?) how old they were when they ventured into and died in freshwater environments, and/or how long they had to live in the ocean to reach their final sizes? Are all the freshwater fossil finds of a similar huge size or might there be different ages/sizes? I am thinking that these fish were far larger than necessary to make the return trip to the lakes and rivers. Probably they were too large to breed in the shallows of smaller rivers and streams.

**Response: We reduced this section and will revisit all of these questions as part of the subject of a future manuscript.**

Overall, I like this paper.

**Thank you!**

Editors comments:
Line 610: please add a reference **added**
Line 626-643, paragraph, discussion on the kype: Please add the published peer revived literature on the subject **Section revised and removed**
Line 738: "fishes" is perhaps not a good term in a scientific paper. Comparing the function of enlarged teeth in groups as distant as elasmobranchs and actinopterygians one could as well discuss elephant tusks. At least elephants and salmonids are both in the same group, osteichthyans. **Noted and revised as “aquatic vertebrates”**
Line 755, please add species name or insert spp. if applies to the entire genus or sp. if the species is unknown **added**
Line 790: please replace "fishes" by a specific scientific term **revised as “actinopterygian and chondrichthyan fishes”**
Line 790-792: axial muscles for raid moth extension, please check if this is correct **Yes, in a study on large mouth bass, axial muscles *“were the primary source of suction expansion power and generated up to 95% of peak expansion power.”* Camp et al. (2015)**
Line 824 and 828: please replace "fishes" by a specific scientific term **“aquatic vertebrates” and “actinopterygians”**
Line 841: spikes or teeth? **“hypertrophied premaxilla and lateral spikes”**
Line 853: add assumed, "assumed zooplanctivory…" **added**
Line 853: "we can reliably rule out" this is a very strong statement, perhaps better down tune a bit **adjusted**
Line 876-868: …"edentulous portion of the dentary, which we interpret as evidence of a kype". Please consider, (a) in Atlantic salmon can have teeth on the kype, (b) in Atlantic salmon teeth are ankylosed to the jaw but the connection to the jaw is not fully mineralised. This was the reason that, based on macerated specimens, an edentulous stage prior to breeding has been described This turned out turned out not to be true, a maceration artefact. There is detailed published literature about the kype and the dentition of Atlantic salmon. **Understood. The concluding statement remains the same, however, earlier discussion is revised.**
Line 871:…"plausible multifunctional". This is speculation that and does not add to the scientific discussion. **Revised**

Journal requirements:

1. Please ensure that your manuscript meets PLOS ONE's style requirements, including those for file naming.

**All files were updated.**

2. In your manuscript, please provide additional information regarding the specimens used in your study. Ensure that you have reported human remain specimen numbers and complete repository information, including museum name and geographic location.

**The section on specimens examined was expanded to include specimens numbers for comparative materials. Acknowledgment for permission to collect on private lands is provided in the text. A statement indicating no permits were required is added to the section on specimens examined. Locality and age were added to section on Systematic paleontology**

3. We note that you have stated that you will provide repository information for your data at acceptance.

**The section on specimens examined was expanded to include specimens numbers for comparative materials and the abbreviates section was expanded to include all institutions from which specimens were examined.**

4. We note that Figures 1, 2, 3, 4, 5, 6, 7 ,8, 9 and S1Fig.tif in your submission contain copyrighted images.

**Co-Author Ray Troll has provided permission to publish his artwork (Strike image and Fig. 1C-D). All other figures are original artwork, CT modeling, and photography by co-author, Claeson.**

5. We are unable to open your Supporting Information file [S1Matrix.nex]. Please kindly revise as necessary and re-upload.

**File is uploaded as a simple text file instead of a nexus file. The same information will be downloadable as a nexus from MorphoBank.org upon publication should future researchers wish to avoid transcribing the dataset themselves.**

6. Please review your reference list to ensure that it is complete and correct.

**Updates were made to citations included in the manuscript as needed when text was modified. The number changes are reflected in the text and can be seen in track changes. No citations were retracted.**
